# Distal versus proximal - an investigation on different supportive strategies by robots for upper limb rehabilitation after stroke: a randomized controlled trial

**DOI:** 10.1186/s12984-019-0537-5

**Published:** 2019-06-03

**Authors:** Qiuyang QIAN, Chingyi Nam, Ziqi Guo, Yanhuan Huang, Xiaoling Hu, Stephanie C. Ng, Yongping Zheng, Waisang Poon

**Affiliations:** 1 Department of Biomedical Engineering, the Hong Kong Polytechnic University, Kowloon, Hong Kong; 2 Department of Surgery, Prince of Wales Hospital, The Chinese University of Hong Kong, Shatin, Hong Kong

**Keywords:** Stroke rehabilitation, NMES-robot, Upper extremity, Supporting strategy

## Abstract

**Background:**

Different mechanical supporting strategies to the joints in the upper extremity (UE) may lead to varied rehabilitative effects after stroke. This study compared the rehabilitation effectiveness achieved by electromyography (EMG)-driven neuromuscular electrical stimulation (NMES)-robotic systems when supporting to the distal fingers and to the proximal (wrist-elbow) joints.

**Methods:**

Thirty subjects with chronic stroke were randomly assigned to receive motor trainings with NMES-robotic support to the finger joints (hand group, *n* = 15) and with support to the wrist-elbow joints (sleeve group, n = 15). The training effects were evaluated by the clinical scores of Fugl-Meyer Assessment (FMA), Action Research Arm Test (ARAT), and Modified Ashworth Scale (MAS) before and after the trainings, as well as 3 months later. The cross-session EMG monitoring of EMG activation level and co-contraction index (CI) were also applied to investigate the recovery progress of muscle activations and muscle coordination patterns through the training sessions.

**Results:**

Significant improvements (*P* < 0.05) in FMA full score, FMA shoulder/elbow (FMA-SE) and ARAT scores were found in both groups, whereas significant improvements (*P* < 0.05) in FMA wrist/hand (FMA-WH) and MAS scores were only observed in the hand group. Significant decrease of EMG activation levels (*P* < 0.05) of UE flexors was observed in both groups. Significant decrease in CI values (*P* < 0.05) was observed in both groups in the muscle pairs of biceps brachii and triceps brachii (BIC&TRI) and the wrist-finger flexors (flexor carpi radialis-flexor digitorum) and TRI (FCR-FD&TRI). The EMG activation levels and CIs of the hand group exhibited faster reductions across the training sessions than the sleeve group (*P* < 0.05).

**Conclusions:**

Robotic supports to either the distal fingers or the proximal elbow-wrist could achieve motor improvements in UE. The robotic support directly to the distal fingers was more effective than to the proximal parts in improving finger motor functions and in releasing muscle spasticity in the whole UE.

**Clinical trial registration:**

ClinicalTrials.gov, identifier NCT02117089; date of registration: April 10, 2014. https://clinicaltrials.gov/ct2/show/NCT02117089

## Introduction

Stroke is one of the leading causes of long-term adult disabilities [1], with rapid growth worldwide [2]. More than 80% of patients suffer from post-stroke motor deficits on their affected upper extremity (UE) [3, 4], and less than 18% of survivors demonstrate near-to-normal functional recovery when measured six months after the onset [5, 6]. Furthermore, a particular challenge for current stroke rehabilitation is that most survivors with chronic stroke still sustain moderate to severe motor impairments in the wrist and hand for daily activities [5, 7], greatly affecting their independence in the daily living [8].

UE motor recovery on the affected side can be effectively promoted through physical training. Significant motor recovery usually occurs within the first six months after the stroke onset [9] and is believed to be plateaued in the chronic period (i.e. six months after stroke onset) [10]. Therefore, rehabilitation resources are usually more concentrated in the early stage than in the chronic period after stroke conventionally. However, more recent studies have reported that repetitive [11] and high-intensity practice [12] can markedly contribute to functional improvement of the affected UE movement, even in patients with chronic stroke [13]. Furthermore, task-oriented training with coordinated practice among different joints in the upper limb has demonstrated to be effective in converting motor improvements into meaningful limb functions for daily activities after stroke [14]. However, it is challenging to manage the coordinated movements with multiple joints (e.g. fingers, wrist, and elbow joints) at the same time in conventional treatments by one-to-one manual operation, which has been further affected by the insufficiency of professional manpower and a short hospital stay even in developed countries [15]. Traditionally, a pair of therapist-patient unit usually starts the training on the larger and more proximal joints and leaves the distal joints being less practiced in the early in-hospital UE rehabilitation, according to the spontaneous motor return after stroke. However, this strategy resulted in the learned non-use in the distal joints and compensatory movements from the proximal in the UE carried over to the chronic period when the distal practice was insufficient after the discharge [16]. New technologies/methods are needed to supplement the labor-demanding and long-term post-stroke physical rehabilitation.

Robots have been useful assistants in intensive and repeated physical treatments for long-term services [17, 18]. Various rehabilitation robotic systems have been developed for specific training purposes and applied to different UE segments [19–24]. However, earlier studies yielded inconsistent findings regarding the training outcomes of robot-assisted therapy. A recent systematic review by Mehrholz and colleagues has also summarized the studies of different robot-assisted upper limb treatments including their training protocols and outcomes [24]. Most studies reported equivalent improvements after robot-assisted training compared with the manual delivered conventional treatments, some of them even indicated better outcomes with the robotic support [25–28], whereas better training effects by conventional manual therapies on the whole upper limb were found when compared with robots with supportive schemes to large and proximal joints with continuous passive motions (CPM) [19, 29]. Previous studies also reported different results in terms of the long-term rehabilitation effects associated with robot-assisted training. For example, Bovolenta and colleagues reported significant improvements in UE motor function right after the robot-assisted training but most of the benefits were lost within three months after a course of the treatment [27]. Meanwhile, the study by Housman and colleagues found that robot-assisted training could not merely carry out significant motor restoration in the UE but also maintain the training effects for at least six months afterwards [30]. Besides the differences in the control design of the robots, one of the major reasons for the diverse rehabilitation effects is the varied mechanical supporting strategies to the UE joints in the training. As reported by Krebs et al., robotic assistance was applied on a single wrist joint but the treatment achieved additional motor improvements in the elbow-shoulder segments, while the elbow-shoulder parts were restricted to move in the training [31]. Similar motor improvements in the proximal joints relative to the target distal joints were also reported by Hu and colleagues when using electromyography (EMG)-driven robots to assist respective physical practices at the fingers and the wrist, and the motor improvements achieved in both the proximal and the distal joints were maintained for three months after the training [32, 33]. The recovery occurring in the proximal joints when the physical training was restricted mainly to the distal joints was primarily due to the competitive interaction between the proximal and the distal joints in physical rehabilitation after stroke and the compensatory muscular activities in the proximal joint when moving the distal [34]. Mechanical supporting strategies could interfere with muscular synergies in the UE during physical training. The varied rehabilitation effects resulting from different joint-supporting strategies have not been adequately investigated yet. In this work, we hypothesized that robotic support to the distal joints would be more effective than to the proximal joints for the whole UE rehabilitation.

In our previous studies, a series of exoskeletal robotic systems [35–38] have been designed for different joints in the UE by using EMG as a bioindicator for the voluntary motor intention from a user. The robots could assist a stroke survivor to conduct UE tasks simulating daily tasks, such as coordinated arm reaching, hand grasping and releasing. It has been proven that the EMG-driven control strategy underpinning our robots was effective for the involvement of voluntary efforts during the training process [19, 39] and could result in more significant motor improvement in the UE than continuous passive motions for chronic stroke [40]. Subsequently, we designed hybrid EMG-driven controls to integrate neuromuscular electrical stimulation (NMES) and robot in one system, i.e., EMG-driven NMES-robots [32, 41, 42]. The related clinical trials suggested that the combined treatment with the respective advantages of NMES and robot could accelerate the rehabilitation progress with a better long-term effect compared with those achieved by robot alone [43]. The purpose of the study was to investigate the training effectiveness of two different joint-supporting strategies by the NMES-robots, i.e. direct support to distal fingers and relatively more proximal support to the wrist-elbow segments, with the same EMG-driven control in UE physical training on chronic stroke patients through a randomized clinical trial. According to the Patient, Intervention, Comparison, Outcome (PICO) Guideline [44, 45], the current study included the following items:

1. Patient: Chronic stroke patients with upper limb dysfunction;
2. Intervention: Administrated with robot-assisted upper limb physical training;
3. Comparison: Direct support to distal fingers and support to relatively more proximal UE segments (i.e. the wrist-elbow parts);
4. Outcome: Training effectiveness of voluntary UE motor function and release of muscle spasticity.

## Methods

### EMG-driven NMES-robots

**Fig. 1:**
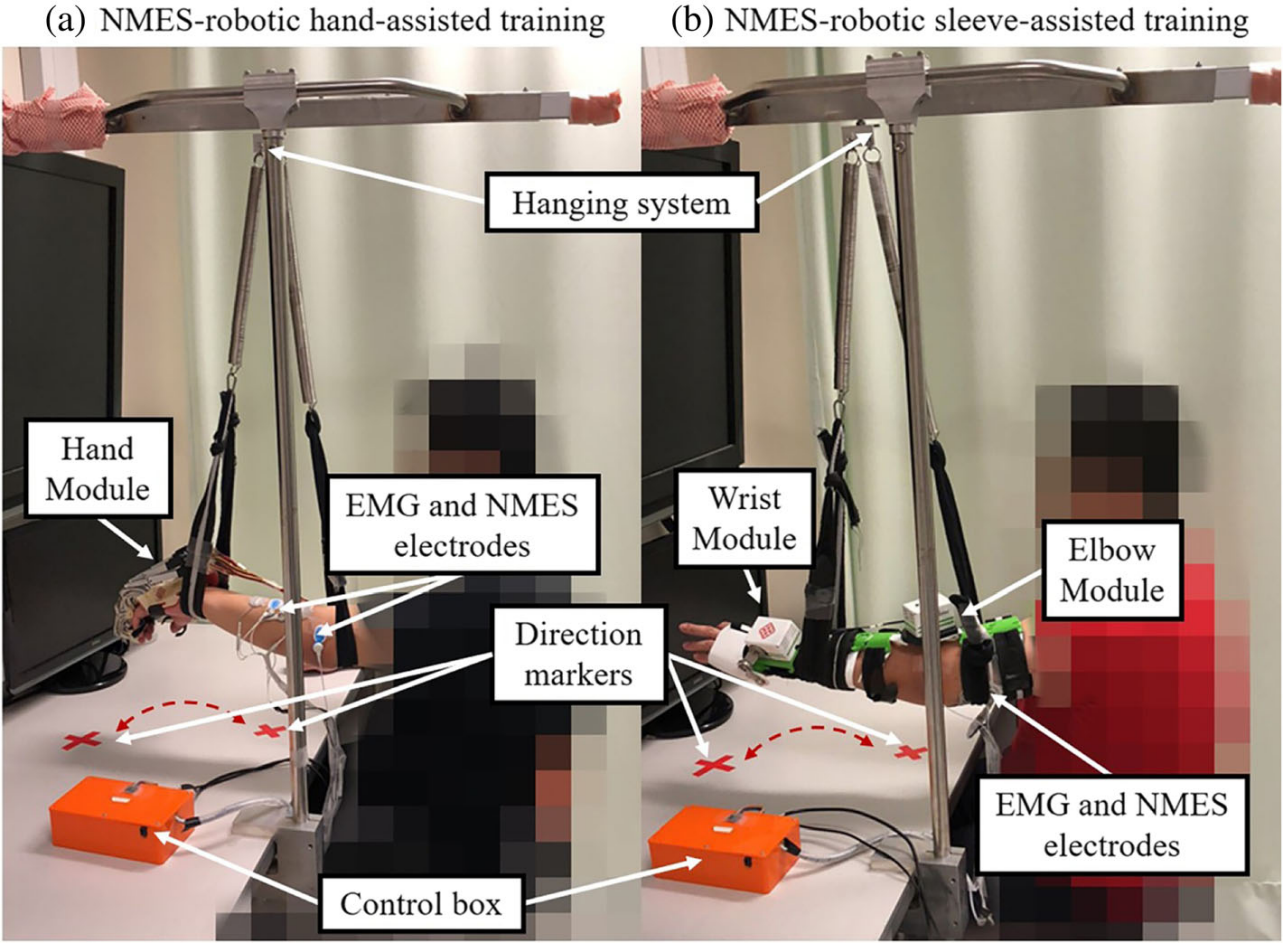
The electromyography (EMG)-driven neuromuscular electrical stimulation (NMES)-robotic system: (**a**) the NMES-robotic hand consisting of a mechanical exoskeleton of the robotic hand, a pair of NMES electrodes attached to the extensor digitorum (ED) muscle, and EMG electrodes on the ED and the flexor digitorum (FD) muscles; (**b**) the NMES-robotic sleeve consisting of a mechanical exoskeleton of the wrist module and elbow module, two pairs of NMES electrodes attached to the extensor carpi radialis (ECR) muscle and the triceps brachii (TRI) muscle, and EMG electrodes on the ECR, flexor carpi radialis (FCR), TRI and biceps brachii (BIC) muscles

The two EMG-driven NMES-robots used in this work were wearable exoskeletons for hand/finger practice (i.e., EMG-driven NMES-robotic hand) and for wrist-and-elbow training (i.e., EMG-driven NMES-robotic sleeve), as shown in Fig. 1a and b.

#### EMG-driven NMES-robotic hand

Figure 1a shows the EMG-driven NMES-robotic hand, which consisted of a palm-wrist module fixed to the wrist and five individual finger assemblies. Each finger assembly was actuated by a linear actuator (Firgelli L12, Firgelli Technologies Inc.) [32]. For the index, the middle, the ring and the little fingers, the proximal section could rotate around the virtual center located at the metacarpophalangeal (MCP) joint, whereas the distal section could rotate around the virtual center located at the proximal interphalangeal (PIP) joint; as regards the thumb, it was designed to rotate around the virtual center of its MCP joint [18]. Each finger assembly could provide a range of motion (ROM) of 55° for the MCP joint and 65° for the PIP joint. One channel NMES electrode pair (30 mm diameter, Axelgaard Corp., Fallbrook, CA, USA) was attached on the skin surface of the extensor digitorum (ED) muscle belly, being capable of providing square pulsed electrical current stimuli with a constant amplitude of 70 V, frequency of 40 Hz, and a manually adjustable pulse width in the range of 0-300 μs (set at the minimum intensity to achieve a fully extended position of the fingers for each individual). No electrical stimulation for finger flexion was used because there is a likelihood of increased spasticity in the flexors in the majority of patients with chronic stroke. The EMG electrode pairs (Blue Sensor N, Ambu Inc., with a contact area of 20 mm × 30 mm) were attached on the skin surface of the muscle bellies of ED and flexor digitorum (FD), with center separation of 2 cm. For the ED muscle, the EMG electrodes were placed perpendicularly to the NMES electrode pair, adopted as an empirical configuration to have relatively low stimulation artifact during EMG signal capturing [46].

An EMG-triggering strategy was adopted for the system control [32, 33], i.e., voluntary EMG from a target driving muscle was only needed to initiate/trigger the system. However, no voluntary muscular effort was needed from a subject, once the robot is initiated. The EMG signals from the target muscles (i.e. FD for finger flexion and ED for finger extension) controlled the assistance from both the robot and the NMES [33]. In each motion phase (i.e. finger flexion or extension), the finger assembly motors would move with a constant velocity (22°/s at MCP and 26°/s at PIP joint) once the EMG activation level of a driving muscle exceeded a pre-set threshold (i.e., three times the standard deviation (SD) above the EMG baseline at rest, by following the standard detection of the onset of voluntary EMG in a contracting muscle [47]). Constant NMES (70 V and 40 Hz) would be only delivered to the ED muscle simultaneously with the motor support in the finger extension phase [32, 33].

#### EMG-driven NMES-robotic sleeve

Figure 1b shows the NMES-robotic sleeve, which consisted of two exoskeleton robotic modules for the wrist and the elbow, respectively [42]. Due to post-stroke joint stiffness and muscle spasticity, the modules were not mechanically connected to ensure that they fitted participants with different ergonomic parameters (e.g. limb length and pronation angles away from the neutral position at the wrist) [48]. Each mechanical module was controlled by an independent servo motor (MX106, ROBOTIS), and would support the joint perform flexion and extension motions with a constant velocity of 10°/s during the training [48]. The orthosis of the wrist module only covered the palm at the hand side and set the fingers free for flexion and extension motions. The maximum ROM provided by the wrist module was from 45° extension to 60° flexion, while for the elbow it was from 30° flexion to 180° extension [41]. Two channel NMES electrode pairs were attached on the muscle bellies of the triceps brachii (TRI) and the extensor carpi radialis (ECR), with the same setting for stimuli parameters (i.e., amplitude, frequency and pulse) as for the NMES-robotic hand training. Moreover, as in the case of the NMES-robotic hand training, electrical stimuli were not delivered to the biceps brachii (BIC) and flexor carpi radialis (FCR) (i.e. the flexors) due to the muscle weakness in the UE extensors and muscular spasticity in the UE flexors for the chronic stroke patients. The EMG electrode pairs were placed on the muscle bellies of BIC, the TRI, the FCR and the ECR. The configuration of EMG and NMES electrodes on the extensors (i.e. TRI and ECR) was the same as that in NMES-robotic hand training.

The control algorithm for the assistance from the robotic sleeve and NMES was the same as the NMES-robotic hand, i.e., once the EMG activation level of a target muscle exceeded 3 times SD above the baseline, the system would be triggered for the related joint motion control (i.e. BIC for elbow flexion, TRI for elbow extension, FCR for wrist flexion and ECR for wrist extension) [42]. NMES was only applied to the extensors.

#### Subject recruitment

**Fig. 2:**
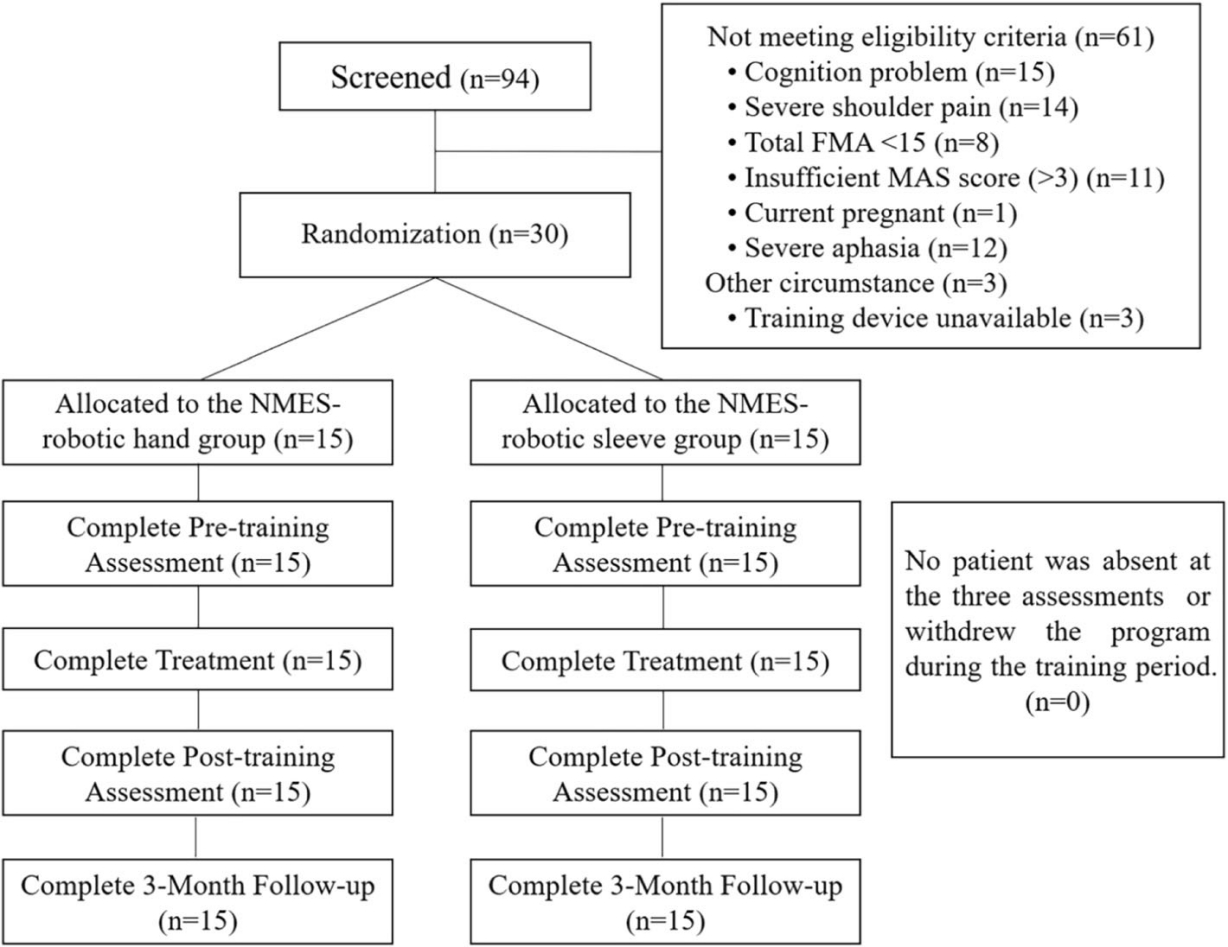
The Consolidated Standards of Reporting Trials flowchart of the experimental design

After obtaining the approval from the Human Subjects Ethics Subcommittee of the university, we screened chronic stroke patients from local districts and then arranged the treatments with the two EMG-driven NMES-robots in a rehabilitation laboratory. The study design was a non-blinded randomized controlled trial with a three-month follow-up (3MFU) for comparing the motor improvements on the upper limb with two different supporting schemes, namely, support to the distal joints (fingers) by EMG-driven NMES-Robotic hand and support to the more proximal joints (wrist-elbow) by EMG-driven NMES-Robotic sleeve. Figure 2 illustrates the Consolidated Standards of Reporting Trials flowchart of the experimental design.

The clinical collaborators of the study screened 94 patients with chronic post-stroke UL motor deficits according to the following inclusion criteria: (1) age range 18–78 years old prior to stroke [49, 50], (2) evidence of acquiring an unilateral brain lesion due to stroke at least six months, without other diagnosed neurological deficits or secondary onset; (3) had enough cognition to understand the content or purpose of the study and follow simple instructions, as assessed by the Mini-Mental State Examination (MMSE> 21) [51], (4) motor impairments affected in the UL ranged from severe to moderate, measured by the Fugl-Meyer Assessment for upper extremity (15 < FMA < 45, with a maximal score of 66) [52]^,^ (5) spasticity affected at the elbow, the wrist and the fingers during enrollment ranged ≤3, as assessed by the Modified Ashworth Scale [MAS, ranged from 0 (no increase in the muscle tone) to 4 (affected part rigid)] [53], (6) had detectable voluntary EMG from the target muscles (i.e., three times SD above the baseline) [32]. If they did not meet the above inclusion criteria or if they were pregnant at the time, had severe dysphasia or had a pacemaker implant, participants were not included. The injection of botulinum toxin (BOTOX) in the upper limb within one year at the time of screening was also one of the exclusion criteria in the study. All the recruited subjects gave consent that they would not receive the BOTOX injection during the whole study period.

Thirty patients who satisfied the inclusion criteria were recruited for this study. They were informed about the research purpose of the study by the project leader and provided their written consents. A recruited participant was randomly allocated into two groups by picking up a masked paper ball from a box without replacement. There were 30 masked paper balls in the box, with 15 marked with ‘1’ to receive the NMES-robot hand training (hand group) and another 15 marked with ‘2’ to receive the NMES-robotic sleeve training (sleeve group). Table 1. shows the demographic and clinical information of the participants after the randomization.

**Table 1:** Demographic data of the participants after the randomization with no significant difference between the two groups (*P* > 0.05): (a) independent t-test; (b) Fisher’s exact test

Characteristics	Training Assisted by NMES-ROBOTS		*P* value
	Hand group (*n* = 15)	Sleeve group (*n* = 15)	
Age (yrs.) ^a^	57.3 ± 8.87	57.7 ± 5.93	0.886
Time since stroke (yrs.)^a^	8.26 ± 4.17	7.87 ± 3.07	0.773
Gender (male/female)^b^	12/3	10/5	0.682
Stroke side (left/right)^b^	7/8	7/8	1.000
Type of stroke ^b^ (hemorrhagic/ischemic)	8/7	6/9	0.715

This work is the first study to compare the training outcomes of two supportive schemes by the NMES-robotic systems in post-stroke upper limb training. The 15 arm design in this work was initially based on our previous single trial study using the NMES-robotic hand on chronic stroke patients [33], where we could observe significant improvements (*p* < 0.05) in FMA-UE and MAS after the training when 15 subjects were recruited. Based on the preliminary results in [33], it showed that 14 subjects were already enough to achieve the significant intragroup difference (5% of type I error with a power of 80% for one-way analysis of variance (1-way ANOVA) [54]). However, there was no previous literature that could provide similar information for the NMES-robotic sleeve group on the chronic stroke. In this work, we assumed that 15 subjects in each group would achieve significant intragroup differences after the treatments, based on the rehabilitation effectiveness of NMES-robots in upper limb rehabilitation on chronic stroke in our previous works [32, 33]. We also assumed that robotic support to the distal joints could achieve better motor improvements than the support to the relatively proximal joints, as hypothesized in the introduction.

#### Training protocol

Both groups received repetitive task-oriented motion practice with participants’ voluntary effort on their entire affected UE, assisted by the two EMG-driven NMES-robots. In this study, all participants were planned to receive 20 sessions of robot-assisted UE training with an intensity of 3–5 sessions/week, at most 1 session/day (1 h for the motion tasks in each session), within a period of 7 consecutive weeks. After the completion of the training, all participants finished the program with a regular attendance, i.e., 4 sessions/week and completed the program in 5 weeks, except one participant completed the program in 7 weeks with a frequency of 3 sessions/week. An average of 180 cycles of the sequenced motion tasks were conducted during the 60-min training in each session for both groups.

#### NMES-robotic hand assisted training

In the beginning, the participants were arranged to sit in front of a table, with their paretic upper limbs suspended by a hanging system (Fig. 1a) supporting at the wrist and elbow joints, in order to offset the gravity effect of the NMES-robotic hand. This design was justified by the fact that most of the participants had difficulty sustaining the weight of both their paretic limbs and the robotic system without support, especially in the first several training sessions. Subsequently, they were required to perform robot-assisted vertical UE training with sequenced and repeated motion tasks according to a visual cue on the screen for a total of 60 min: (1) elbow extension in forward reaching, (2) wrist extension and hand open, (3) wrist flexion and hand close, and (4) elbow flexion (withdrawing). To prevent muscle fatigue, participants were allowed to rest for 10 min after half an hour of training [33]. If the participants could not reach out at the elbow in the initial sessions, they were encouraged to try their best to complete the motion tasks.

#### NMES-robotic sleeve assisted training

During the sleeve-assisted training, the paretic upper limbs of the participants were also suspended by the hanging system (Fig. 1b) to resist the gravity effect of the NMES-robotic system. The training task for the sleeve group was the same as that for the hand group, including the sequential motion tasks, i.e. (1) elbow extension in forward reaching, (2) wrist extension and hand open, (3) wrist flexion and hand close, and (4) elbow flexion (withdrawing), as prompted by the visual cues on the computer screen. Each training session lasted for a total of 60 min, with an extra 10-min break between two consecutive 30-min intervals to avoid muscle fatigue [42].

The main objective of the motion tasks was to simulate arm reaching-grasping and withdrawing motions in daily activities. Markers on the table (Fig. 1) were labelled for the participants to recognize the targeting positions of the hand in the horizontal plane during the motions.

### Outcome evaluation

#### Clinical assessments

In this study, all participants underwent clinical assessments before, after training and three months later. The FMA for upper extremity (FMA-UE, full score 66) was used to evaluate the performance-based sensorimotor functions of the paretic upper limbs. Furthermore, to compare the motor functions between the proximal and distal segments, the FMA was sub-scaled into shoulder/elbow (42/66) and wrist/hand (24/66). The Action Research Arm Test (ARAT) was adopted mainly to evaluate motor functions with hand tasks, including holding/releasing objects in different shapes, sized and weights. Moreover, post-stroke spasticity at the fingers, the wrist and the elbow were assessed by applying the MAS. All the clinical assessments were conducted by a collaborative physiotherapist who was blinded to the group information. Communication between the participants and the assessor regarding training details were not allowed in the study. Normality tests by Lilliefors method [55] were conducted on all clinical scores. They obeyed the normal distribution (*P* > 0.05). The score of ‘1+’ in the MAS was converted to 1.4 in this work as practiced in our previous studies [33, 42] and in the literature [56, 57] for numerical calculation.

#### EMG measurement

In addition to the clinical assessments, session-by-session EMG evaluation before the device-assisted training was used to trace the evolution of the muscle coordination and the recovery progress of each target muscle across the 20 training sessions with maximum voluntary contractions (MVC) and a bare arm test, as practiced previously [46, 58]. The test was similar to the motion tasks in the formal training but without support by NMES-robot, consisting of horizontal arm reaching, hand grasping, hand opening, and arm withdrawing tasks, and was repeated three times [32, 33, 41, 42]. EMG signals from BIC, TRI, ECR-ED unit, and FCR-FD unit were collected for off-line processing. In the context of the investigation of EMG activities in the forearm for both groups, the EMG electrode pairs were located on the common area of the two muscle bellies of ECR-ED and FCR-FD due to the close anatomical proximity between the ECR and ED muscles and between the FCR and FD muscles. All EMG signals were amplified with a gain of 1000 (amplifier: INA 333, Texas Instruments Inc.), band-pass filtered from 10 to 500 Hz, and then sampled with 1000 Hz for digitization, as was done previously [33, 42].

Two EMG parameters were adopted for quantitative description of the cross-session variations in (1) muscle activation (normalized EMG activation level of each muscle) and (2) muscle coordination pattern (normalized co-contraction index, CI between the muscle pairs). The EMG raw data from the MVCs and bare-arm test helped to calculate the EMG activation levels [42], and the CI between a pair of muscles could be expressed as:

1 $$ \mathrm{CI}=\frac{1}{T}{\int}_0^T{A}_{ij}(t) dt, $$

where *A*_*ij*_*(t)* represented the overlapping activity of EMG linear envelopes for muscle *i* and *j*, and *T* was the length of the signal [42]. Increase in CI values was potentially indicative of aggravation of muscle coordination patterns of a muscle pair with broadened overlapping area, while a decrease in CI values was indicative of separation in the co-contraction phase of the two muscles with the reduced overlapping area.

In this study, a further normalization was applied to both EMG parameters (EMG activation level and CI) of individual participants, with respect to the maximal and minimal values of the participants across the 20 training sessions. The purpose of this procedure was to illustrate the tendency of EMG parameters of an individual with normalized values to vary from 0 to 1 and to minimize the variations among different participants, as encountered previously [35, 36].

### Statistical analysis

The two groups were examined for baseline differences by using independent t-test or Fisher exact tests for their demographic data (*P* > 0.05, Table 1 ). The two groups did not differ significantly in the baseline of all clinical scores (i.e., pre-assessments on FMA, ARAT and MAS, *P* > 0.05, independent t-test, Table 2). The results of clinical assessments were first analyzed using the two-way analysis of covariance (2-way ANCOVA), with respect to the factors of 1) treatment (i.e. NMES-robotic hand training and sleeve training) and 2) the evaluation time point, i.e., the pre-, the post-, and the three-month follow-up assessments, by taking the pre-assessment as a covariate, in order to further minimize the possible baseline difference between the groups [59]. When a significant difference with respect to the time points was found, 1-way ANOVA was conducted to determine the intra-group differences. Subsequently, the between-group comparisons on the clinical scores at the respective post- and 3MFU were evaluated by one-way analysis of covariance (1-way ANCOVA) with the pre-assessment as a covariate. It was not necessary to use the initial EMG parameters (i.e. EMG activation level and CI values) as a covariate for ANCOVA, mainly due to the normalization mentioned above and also due to the fact that the initial values were usually the peak among the 20 training sessions. Two-way analysis of variance (2-way ANOVA) was first applied for the EMG parameters with respect to the group factor and the factor of training times (i.e. 20 sessions). Subsequently, 1-way ANOVA was performed to investigate the variation across the 20 training sessions. If significant group difference was found by 2-way ANOVA with respect to the group factor, independent t-test would be applied at different training sessions for the investigation of intergroup differences. The initially accepted alpha for statistical significance was set at 0.05 in this study. The significant levels at 0.01 and 0.001 were also indicated in Table 2 and Table 3. All statistical calculation in the study was conducted by SPSS 24.0 (2016). Bonferroni corrections were adopted in the post hoc tests in the 1-way ANOVAs. The final *P* value for assessing all the clinical scores was 0.05/3 and that for the cross-sessional EMG parameters was 0.05/20, which were automatically corrected in the SPSS toolbox. In this study, the FMA and MAS clinical scores were the primary outcomes; and the ARAT scores and EMG parameters were the secondary outcomes. It was because that FMA reflected task-specified voluntary motor functions in the whole upper limb and could further investigate the variation in both distal and proximal UE segments by its sub-scales. The MAS could measure different levels of muscle tone and reflect the variation of muscle spasticity [53, 56], which is another major problem impeding UE movements in chronic stroke patients, besides the motor impairment assessed by FMA.

**Table 2:** The means and 95% confidence intervals for each measurement of the clinical assessments, and the probabilities with the estimated effect sizes of the statistical analyses.

Assessment	PRE	POST	3MFU	1-way ANOVA	2-way ANCOVA		
					*P* (*Partial η*^*2*^)		
	Mean Value (95% Confidence Interval)			*P* (*Partial η*^*2*^)	*Session*	*Group*	S*G
FMA Full (Hand)	28.9	42.2	45.3	0.001^###^ (0.274)	0.000^ΔΔΔ^(0.567)	0.879 (0.000)	0.920 (0.002)
	(22.6–35.1)	(35.9–48.5)	(39.0–51.5)				
FMA Full (Sleeve)	32.4	44.8	47.5	0.004^##^ (0.229)			
	(25.9–38.9)	(38.3–51.3)	(41.0–54.0)				
FMA-SE (Hand)	20.0	28.5	30.6	0.001^###^ (0.270)	0.000^ΔΔΔ^(0.550)	0.793 (0.001)	0.825 (0.005)
	(16.0–24.0)	(24.4–32.5)	(26.6–34.6)				
FMA-SE (Sleeve)	21.7	30.7	31.5	0.001^###^ (0.271)			
	(17.8–25.6)	(26.8–34.6)	(27.6–35.4)				
FMA-WH (Hand)	8.9	13.7	14.7	0.005^##^ (0.222)	0.000^ΔΔΔ^(0.362)	0.695 (0.002)	0.698 (0.009)
	(6.3–11.4)	(11.2–16.3)	(12.1–17.2)				
FMA-WH (Sleeve)	10.7	14.1	16.1	0.075 (0.116)			
	(7.3–14.0)	(10.8–17.5)	(12.7–19.4)				
ARAT (Hand)	15.6	26.5	26.9	0.036^#^ (0.147)	0.000^ΔΔΔ^ (0.396)	0.430 (0.080)	0.938 (0.002)
	(8.8–22.4)	(19.7–33.3)	(20.1–33.7)				
ARAT (Sleeve)	20.8	31.9	33.3	0.034^#^ (0.149)			
	(13.6–28.0)	(24.7–39.1)	(26.1–40.5)				
MAS-elbow (Hand)	1.5	0.9	0.7	0.033^#^ (0.149)	0.000^ΔΔΔ^(0.191)	0.591 (0.003)	0.388 (0.023)
	(1.1–2.0)	(0.4–1.3)	(0.3–1.2)				
MAS-elbow (Sleeve)	1.1	0.8	0.7	0.288 (0.058)			
	(0.7–1.5)	(0.4–1.2)	(0.3–1.0)				
MAS-wrist (Hand)	1.5	0.6	0.3	0.001^###^ (0.295)	0.000^ΔΔΔ^(0.518)	0.000^ΔΔΔ^ (0.319)	0.001^ΔΔΔ^ (0.149)
	(1.1–1.9)	(0.2–1.0)	(−0.1–0.7)				
MAS-wrist (Sleeve)	1.3	0.9	0.9	0.272 (0.060)			
	(0.9–1.8)	(0.5–1.4)	(0.4–1.3)				
MAS-finger (Hand)	1.3	0.5	0.4	0.004^##^ (0.231)	0.000^ΔΔΔ^ (0.367)	0.001^ΔΔΔ^ (0.136)	0.067 (0.063)
	(0.9–1.7)	(0.0–0.9)	(0.0–0.8)				
MAS-finger (Sleeve)	1.4	1.0	0.9	0.319 (0.053)			
	(0.9–1.9)	(0.5–1.5)	(0.5–1.4)				

Differences with statistical significance are marked with superscripts beside the *P* values (‘#’ for 1-way-ANOVA intragroup tests, ‘Δ’ for 2-way ANCOVA tests on the group and session effects with the pre-assessment as the covariate). Significant levels are indicated as, 1 superscript for < 0.05, 2 superscripts for ≤0.01, and 3 superscripts for ≤0.001

**Table 3:** The statistical probabilities and the estimated effect sizes of the 1-way analysis of covariance (ANCOVA) on the respective post-assessment and 3-month follow-up (3MFU) between the groups, by taking the pre-assessment as the covariate

Assessment	1-way ANCOVA on the Post- and 3MFU assessments between the groups	
	*Post*_*_Pre*_ P (*Partial η*^*2*^)	*3MFU_*_*Pre*_ P (*Partial η*^*2*^)
FMA		
Full Score	0.808 (0.002)	0.9090 (0.001)
Shoulder/Elbow	0.601 (0.010)	0.601 (0.010)
Wrist/Hand	0.996 (0.000)	0.8070 (0.002)
ARAT	0.721 (0.005)	0.458 (0.021)
MAS		
Elbow	0.686 (0.006)	0.661 (0.007)
Wrist	0.218 (0.054)	0.000^***^ (0.557)
Finger	0.003^**^ (0.289)	0.008^* *^ (0.234)

Differences with statistical significance are marked with ‘*’ beside the *P* values. Significant levels are indicated as, * P < 0.05, ** P ≤ 0.01, *** P ≤ 0.001

## Results

The UE training assisted by NMES-robot was completed by all of the recruited participants, either by using the NMES-robotic hand (*n* = 15) or the NMES-robotic sleeve (*n* = 15). Table 2 summarizes all clinical scores measured in this study, namely, the means and 95% confidence interval of each clinical assessment together with the 1-way ANOVA probabilities with the effect sizes (EFs) for the intra-group evaluation with respect to the assessment sessions, and the 2-way ANCOVA probabilities with EFs with respect to session and group. Table 3 summarizes the probabilities and EFs of the between-group comparison on the respective post- and 3MFU assessments by 1-way ANCOVA with the adjustment of the baseline effect. We compared the demographic data between the groups by independent t-test or Fisher exact test as shown in Table 1. The initial motor status between the groups was also compared with the full score of FMA on the upper limb by independent t-test, as well as with other clinical scores in Table 2. No significant differences in the baselines were observed between the two groups.

### Clinical score

**Fig. 3:**
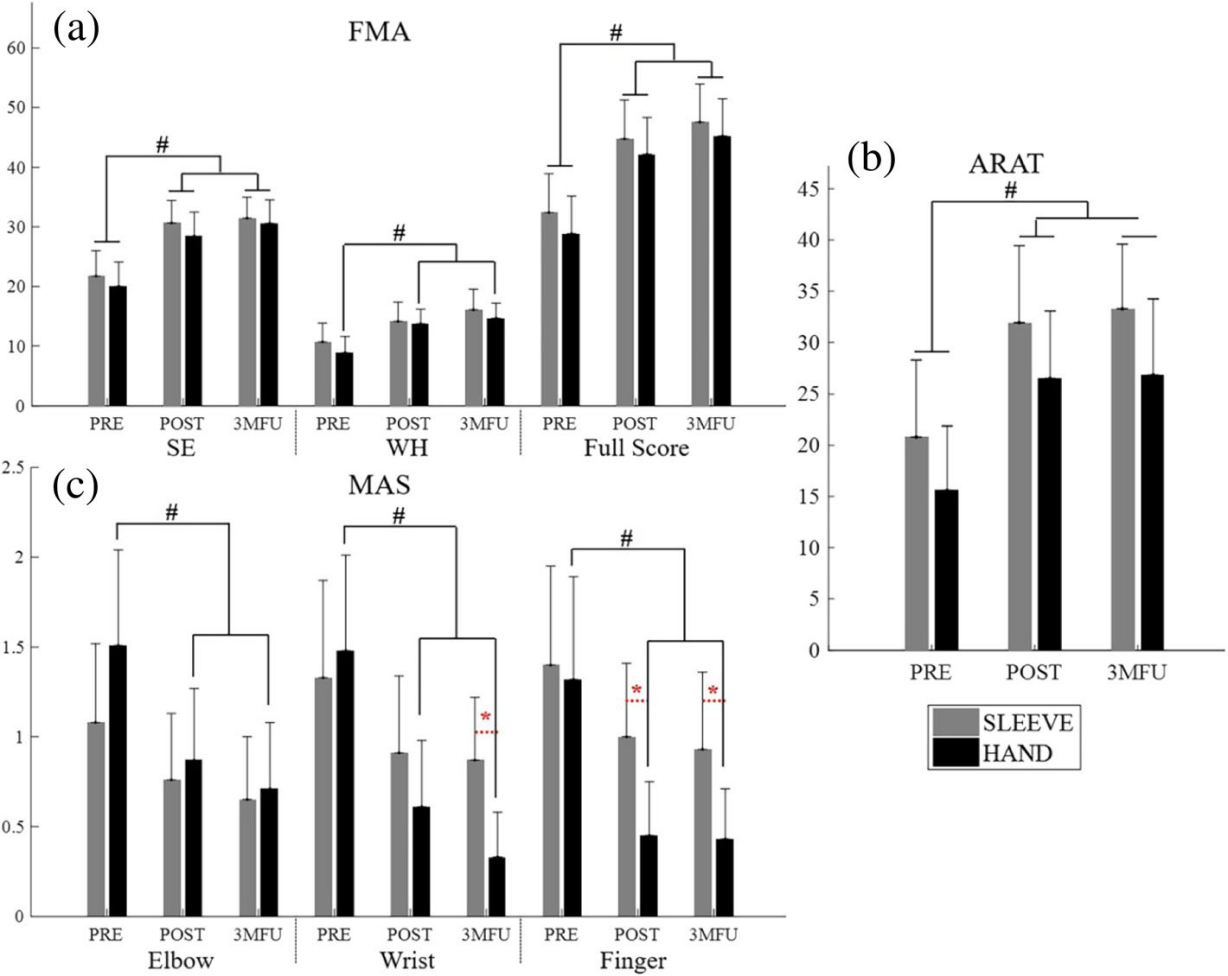
The clinical scores, evaluated before the first (pre-assess) and after the 20th training session (post-assess), as well as the 3-month follow-up (3MFU), of the participants in both NMES-robotic hand and sleeve groups: (**a**) Fugl-Meyer Assessment for the upper limb, FMA full score, FMA shoulder/elbow scores (FMA-SE), and FMA wrist/hand scores (FMA-WH); (**b**) Action Research Arm Test (ARAT) scores; (**c**) Modified Ashworth Scale (MAS) scores at the elbow, the wrist, and the fingers, presented as mean value with 2-time SE (error bar) in each evaluation session. The grey bars are for the sleeve group, and the black bars are for the hand group. The significant inter-group difference is indicated by the ‘*’ [*P* < 0.05, one-way analysis of covariance (ANCOVA)], and ‘#’ is used to indicate the significant intragroup difference [*P* < 0.05, one-way analysis of variance (ANOVA) with Bonferroni post hoc tests]

The FMA scores varied with respect to the whole upper limb as well as to distal and proximal segments, as shown in Fig. 3a. Significant difference was observed only with respect to the factor of evaluation time points in the FMA full score, the FMA shoulder/elbow (FMA-SE) and FMA wrist/hand (FMA-WH) sub-scales (2-way ANCOVA, *P* < 0.05, Table 2). By contrast, no significant difference was observed with respect to the factor of groups. After the training, the FMA full score of both groups exhibited significant increment (hand group: *P* = 0.001, EFs = 0.274, 1-way ANOVA with Bonferroni post hoc test; and the sleeve group: *P* < 0.005, EFs = 0.229, 1-way ANOVA with Bonferroni post hoc test, Table 2), as did the FMA-SE score (hand group: *P* = 0.001, EFs = 0.271, 1-way ANOVA with Bonferroni post hoc test; and the sleeve group: *P* = 0.001, EFs = 0.271, 1-way ANOVA with Bonferroni post hoc test, Table 2). The sleeve group did not display the significant intragroup difference in terms of the FMA-WH score, while a significant increase was observed in the hand group (*P* < 0.01, EFs = 0.222, 1-way ANOVA with Bonferroni post hoc test, Table 2).

Figure 3b presents the ARAT scores in the pre-, post-training and three-month follow-up assessment. A significant difference was observed with respect to the evaluation time points (*P* < 0.001, EF = 0.396, 2-way ANCOVA, Table 2), whereas no significant difference was observed with respect to the groups. Furthermore, after the training, both groups exhibited significant increment compared to the pre-training values, and the elevation was maintained until three months later when evaluation was repeated (hand group: *P* < 0.05, EFs = 0.147, 1-way ANOVA with Bonferroni post hoc test; and the sleeve group: *P* < 0.05, EFs = 0.149, 1-way ANOVA with Bonferroni post hoc test, Table 2).

Figure 3c indicates the variation in MAS scores at the elbow, wrist, and the finger across the evaluation sessions for the two groups. Significant differences were observed with respect to the evaluation time points by 2-way ANCOVA at elbow (*P* < 0.001, EFs = 0.191, Table 2), wrist (P < 0.001, EFs = 0.518, Table 2) and fingers (*P* < 0.001, EFs = 0.367, Table 2). Significant differences with respect to the groups were detected by 2-way ANCOVA at the wrist (*P* < 0.001, EFs = 0.319, Table 2) and finger joints (*P* < 0.001, EFs = 0.136, Table 2). A significant interaction between the factors of the group and the evaluation time point was captured at the wrist (*P* < 0.001, EFs = 0.149, 2-way ANCOVA, Table 2). Through the three evaluation time points, the hand group showed significantly decreased MAS scores at the elbow (*P* < 0.05, EFs = 0.149, 1-way ANOVA with Bonferroni post hoc tests, Table 2), the wrist (*P* < 0.001, EFs = 0.295, 1-way ANOVA with Bonferroni post hoc tests, Table 2), and the fingers (*P* < 0.01, EFs = 0.231, 1-way ANOVA with Bonferroni post hoc tests, Table 2). By contrast, the sleeve group did not reveal any significant intragroup difference at any of the three parts (i.e. elbow, wrist and fingers) in terms of the MAS scores. In the between-group comparison of MAS scores, values in the hand group were significantly lower than those in the NMES-robot sleeve group at fingers in the post-training assessment (*P* < 0.01, EFs = 0.289, 1-way ANCOVA, Table 3) and 3MFU assessment (*P* < 0.01, EFs = 0.234, 1-way ANCOVA, Table 3), while at wrist, the two groups were significantly different only in terms of the 3MFU assessment (*P* < 0.001, EFs = 0.557, 1-way ANCOVA, Table 3).

### EMG parameters

**Fig. 4:**
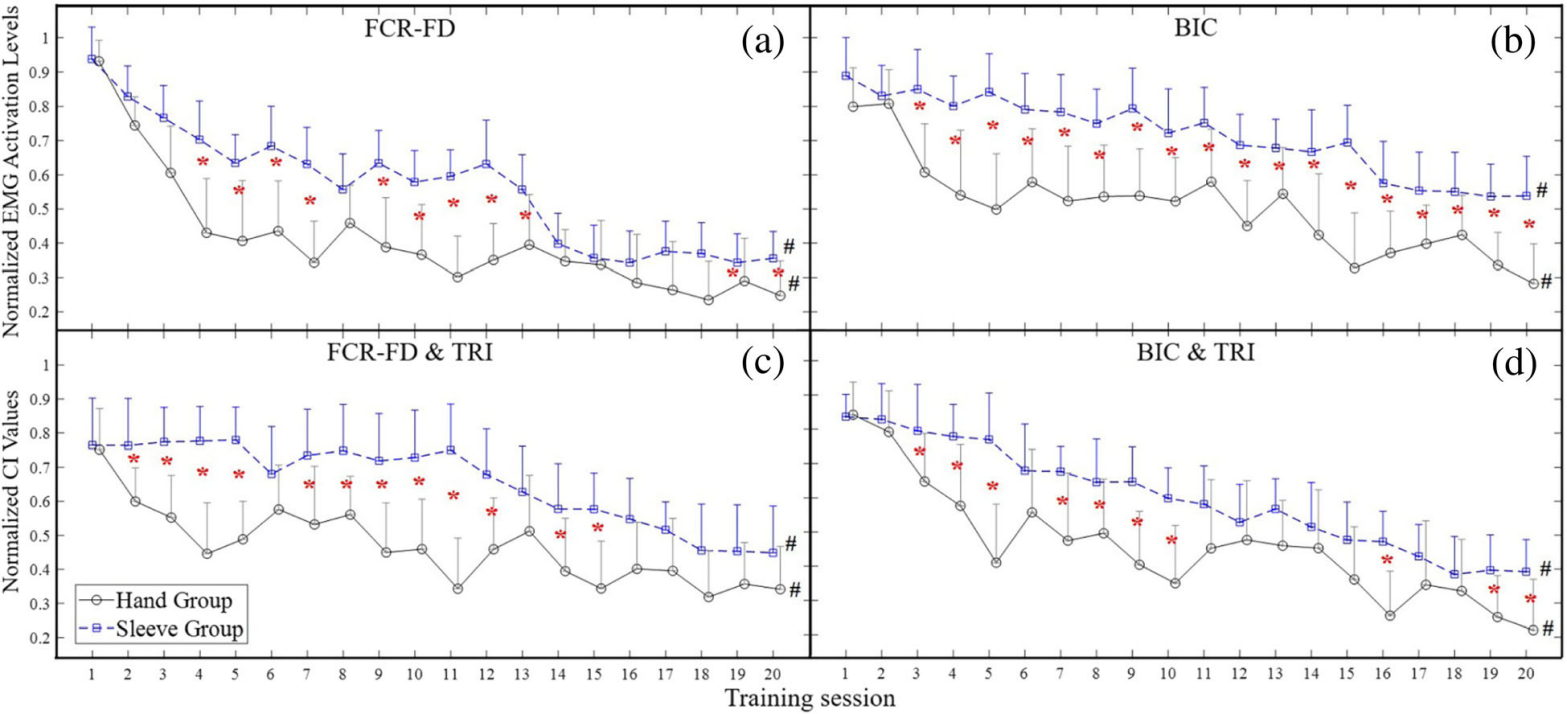
The variation of electromyography (EMG) parameters recorded across the 20 training sessions: (**a**) the changes of the normalized EMG activation levels with significant decline observed in the FCR-FD muscle union (*P* < 0.05, 1-way ANOVA with Bonferroni post hoc tests) in both the hand group and sleeve group; (**b**) the significant decline of the normalized EMG activation levels in the BIC muscle (*P* < 0.05, 1-way ANOVA with Bonferroni post hoc tests) in both groups; (**c**) the significant decline of the normalized co-contraction indexes (CI) values observed in the FCR-FD&TRI muscle pairs (*P* < 0.05, 1-way ANOVA with Bonferroni post hoc tests); (**d**) the changes of CI values with significant decrease in the BIC&TRI muscle pairs (P < 0.05, 1-way ANOVA with Bonferroni post hoc tests). The values are presented as mean value with 2-time SE (error bar) in each session. The solid lines are for the hand group, and the dashed lines are for the sleeve group. The significant inter-group difference is indicated by ‘*’ (*P* < 0.05, independent t-test) for each session, and ‘#’ is used to indicate the significant intragroup difference across the 20 training sessions

Figure 4a to d demonstrate the variation patterns of EMG parameters (i.e. the normalized EMG activation levels and the normalized CI values) across the 20 training sessions in both the hand group and sleeve group. Significant group differences have been found in the illustrated four parameters (2-way ANOVA, *P* < 0.05). Figure 4a indicates that, from the fourth training session, the hand group exhibited significantly lower EMG activation values of FCR-FD muscle union (*P* < 0.05, t-test). Moreover, the values of BIC were also significantly lower in the hand group (*P* < 0.05, t-test) from the third training session and remained lower until the twentieth session, as shown in Fig. 4b. Both groups exhibited significant decrease in the EMG activation level at the FCR-FD muscle union (hand group: *P* < 0.05, EFs = 0.436, 1-way ANOVA with Bonferroni post hoc test; and sleeve group: *P* < 0.05, EFs = 0.151, 1-way ANOVA with Bonferroni post hoc test) and the BIC muscle (hand group: *P* < 0.05, EFs = 0.375, 1-way ANOVA with Bonferroni post hoc test; the sleeve group: *P* < 0.05, EFs = 0.112, 1-way ANOVA with Bonferroni post hoc test). As regards the between-group comparison.

Figure 4c and d demonstrate the variation patterns of CI values across the 20 training sessions. In terms of the between-group comparison, the hand group exhibited significantly lower CI values (P < 0.05, t-test) than the sleeve group from the second to the fifteenth training session in the FCR-FD & TRI muscle pair (Fig. 4c). Besides, the hand group had significantly lower CIs from the third to the twentieth training session in the BIC & TRI muscle pair (Fig. 4d). Additionally, a significant decrease in CI values was observed in both groups in the muscle pairs FCR-FD&TRI (hand group: *P* < 0.05, EFs = 0.185; and sleeve group: *P* < 0.05, EFs = 0.156, 1-way ANOVA with Bonferroni post hoc test) and BIC&TRI (hand group: *P* < 0.05, EFs = 0.301; and sleeve group: *P* < 0.05, EFs = 0.168, 1-way ANOVA with Bonferroni post hoc test).

Regarding the variation patterns of CIs of both the FCR-FD&TRI and the BIC&TRI muscle pairs, the CIs gradually declined and did not reach a plateau over the 20 training sessions. There was no significant increment or decrease in the EMG parameters observed in other target muscles or muscle pairs.

## Discussion

The study compared two different mechanical supporting schemes for UE rehabilitation in chronic stroke by using the EMG-driven NMES-robots, namely, support to the elbow and wrist versus support to the fingers. The results obtained revealed that the two training schemes with different supporting strategies led to UE motor recovery measured by the clinical scores and session-by-session evaluated EMG parameters in all participants. The training tasks for the two groups were same involving the whole upper limb for the reaching and withdrawing tasks, although the two groups were supported at either proximal or distal segments in the upper limb with the NMES-robotic systems. We expected that the motor improvements could be obtained in the whole upper limb rather than in a single segment for the groups. The FMA-UE was used to evaluate the changes of voluntary motor function in the whole upper limb. Its sub-scales (i.e., FMA-WH and FMA-SE) could provide information about the sub-segments in the limb. Furthermore, the release of muscle spasticity at different joints was evaluated by MAS, which was triggered by velocity-dependent passive motions [60, 61] and ARAT was applied to assess the hand related motor restoration [62, 63].

### Motor outcomes evaluated by clinical scores

The increase of FMA score and its sub-scales demonstrated the voluntary motor improvements achieved by the two different joint supporting strategies, as well as the improvements in the related UE segments, namely, distal (wrist-hand) and proximal (elbow-shoulder) parts. Both supporting strategies significantly improved the overall UE motor functions after the training. We also noticed that, compared to pre-assessment, the averaged FMA full scores in robotic hand group increased by 46.1% right after the treatment (post-assessment) and by 56.8% at three-month follow-up, when the ratio was 38.2% (post-assessment) and by 46.7% (3MFU) in the robotic sleeve group. This suggested that motor improvements continued in both groups over a period of three months after treatment completion. For the FMA-SE, the average scores increased by 42.4% (post-assessment) and 54% (3MFU) in the robotic hand group, with the ratio of 41.1% (post-assessment) and by 44.8% (3MFU) in the robotic sleeve group. The motor improvements in the FMA-SE subscale for the robotic hand group confirmed that robotic support at the distal fingers could also benefit the proximal joint recovery (i.e., shoulder/elbow), similar to the observations reported in the literature [31, 64]. The motor gain achieved in the robotic hand group was comparable to that for the sleeve group where direct robotic supports were provided to the proximal joints. In this work, proximal improvements in the robotic hand group were related to the compensatory contraction of proximal UE muscles during the recruitment of distal muscles in NMES-robotic hand training and the competitive interaction between distal and proximal muscles during the sequenced motion tasks, as mentioned earlier [34]. For the evaluation on the distal UE by FMA-WH subscale, the average scores increased by 54.8% in the robotic hand group and by 32.4% in the robotic sleeve group at post-assessment. A further increase by 65.4% in the robotic hand group and by 50.6% in the sleeve group was reported at three-month follow-up. However, significant improvement across three evaluation time points (i.e., pre-assess, post-assess and 3MFU) at wrist-hand was achieved only in the robotic hand group and not in the sleeve group, as shown in Fig. 3. The results suggested that direct support to the finger joints was more effective to achieve distal motor improvements than support to more proximal (i.e., wrist-elbow) joints, and the improvement could continue in the three months after the training.

The improvements in the ARAT scores were consistent with the observations obtained by FMA scores. The ARAT results suggested that both treatments could improve the voluntary motor functions in the whole upper limb, with an emphasis on daily tasks involving finger functions. The improvement for both groups could last for three months after the training. Although FMA-WH improvement was not significant for the robotic sleeve group, the significant improvements in the ARAT also suggested the distal improvements achieved by the sleeve training. However, besides evaluating hand grasping and fingers gripping functions, the ARAT assessments tested the positioning of extremities and the choice of objects with varied weights as well. These evaluation items were related to the motor function of proximal UE segments [62], which could benefit from the treatment of the wrist-elbow parts. Furthermore, we found increased scores in the subscale items of lifting UE and placing hand to various pericranial positions in both groups, although the robotic sleeve group achieved higher scores. This was due to the fact that ARAT uses a specific time limit to define the level of deficits [63]. The between-group differences in the items were not significant but higher scores showed a trend of better smoothness of the movements after training by the NMES-robotic sleeve.

The MAS scores showed the descending trend of muscle tone in both groups by supporting different UE segments. Significant between-group differences obtained at the wrist (only in the 3MFU) and at the fingers (in both the post-assessment and 3MFU) demonstrated the markedly declined muscle tone in the robotic hand group. The use of the NMES-robotic hand led to a significant release of muscle spasticity, which could be maintained for three months after training. Meanwhile, the decline of muscle tone was not significant in the robotic sleeve group according to the MAS scores at all three parts (i.e. the fingers, the wrist and the elbow). The MAS results suggested that direct robotic assistance at the finger joints could more effectively release the spasticity at the distal. One possible reason for the better performance in MAS of the whole UE in the robotic hand group was that the participants exerted more voluntary effort in the arm-reaching tasks than the sleeve group when the elbow and wrist were not actuated. Maximized involvement of voluntary effort in post-stroke limb practice has been found to be an important factor related to the significant release of muscle tone with long-term effects [35]. Furthermore, it was common that persons with chronic stroke had better proximal limb functions than the distal. When the distal joints (e.g., the fingers in this work) were assisted by the NMES-robotic hand to perform the tasks they could not achieve (e.g., hand open) by themselves, they would be promoted to practice.

### Motor outcomes evaluated by EMG parameters

The session-by-session EMG evaluation demonstrated the recovery progress in the muscle coordination across the 20 training sessions for both groups, by monitoring the activation and coordination patterns among the four individual muscles/muscle unions (i.e. BIC, TRI, FCR-FD and ECR-ED).

In this work, the EMG activation level of FCR-FD muscle union (flexors in the distal UE segments, i.e. fingers and wrist) and BIC muscle (a flexor in the more proximal UE, i.e. elbow) in the hand group tended to decrease more rapidly than those in the sleeve group across the training process, as shown in Fig. 4. In the hand group, FCR-FD and BIC decreased rapidly by 50 and 32%, respectively, over the first four sessions, and decreased by a further 19 and 31.9%, respectively, from the fifth to the twentieth sessions. By contrast, the sleeve group showed a gradual decrease by 50% (FCR-FD) over 14 sessions and by 32% (BIC) over 16 sessions. The results not only suggested the reduced spasticity of the related joints in both groups [65], but also implied that the release of spasticity in the entire UE was more effective by supporting to the distal joints (i.e. fingers) than to the more proximal parts (i.e. wrist-elbow). The EMG observation was consistent with the variation of MAS scores in the elbow, the wrist and the fingers for both groups, which manifested the differences between the two NMES-robot supportive schemes in the upper limb rehabilitation after stroke. Furthermore, the decrease in the EMG activation level could also be attributed to the reduction in excessive muscle activities of FCR-FD and BIC muscles during the bare arm test for arm reaching, withdrawing and hand grasping motions [66]. The faster decrease of EMG activation levels by supporting the distal UE segments could be a reason for better performance in the FMA scores and its subscales for patients in the hand group.

The CI values revealed the coactivity of a muscle pair, either within one joint or across joints. Compared to the sleeve group, the hand group exhibited fasted reduction of CI values in FCR-FD&TRI and BIC&TRI. With supporting to the distal segments during the training, CI values associated with FCR-FD&TRI decreased rapidly by 40.7% over the first four training sessions, while the CI values associated with FCR-FD&TRI declined by 40.3% over 19 training sessions. The CI values in the hand group were significantly lower than those in the sleeve group through the first 15 training sessions. As for BIC&TRI, the CIs also decreased more rapidly in the group with support to the distal UE than with support to more proximal parts. The values decreased by 51% over the first five training sessions in the hand group but decreased only by 7.9% at the same evaluation point (5th session) in the sleeve group. As no significant change was found in the TRI, the reduction of FCR-FD and BIC muscle activation level was related to the decrease in the CI values of FCR-FD&TRI and BIC&TRI. The muscles associated with both proximal and distal joints commonly exhibited excessive co-contractions after stroke [67]. The significant reduction of CI values in FCR-FD&TRI indicated the release of their co-contraction patterns and implied the improved isolation of the distal joint (i.e. wrist) movements from the more proximal joint (i.e. elbow). The improvements could reflect evolutionary and more independent motion patterns during the bare arm test and clinical assessments of ARAT and FMA. Meanwhile, the significant decrease of CI values in BIC&TRI showed the release of co-contraction patterns in the elbow joint and indicated the promotion of arm reaching and withdrawing movements through elbow extension and flexion. Compared to the provision of support to the more proximal parts, provision of support to the distal joints could lead to a more effective improvement in the release of muscle co-contraction during the UE rehabilitation.

In the study, we noticed that the recovery process did not reach a plateau within the 20 training sessions with the acceleration of EMG activation levels in the FCR-FD and BIC for both groups, and similar patterns could be found in the CIs of the FCR-FD&TRI and BIC&TRI in both groups as well. In an earlier study, it was suggested that a plateau of little or no change in performance was indicative of the fact that learning of a skilled movement had come to an end [68]. Hence, the results of EMG parameters could suggest that further improvement in the recovery of the upper limb at both distal and proximal segments could be obtained through additional training. In the work by Dewald and colleagues [69, 70], it was shown that decreasing the burden on the shoulder girdle musculature was associated with more independent UE joint control with long-term effects. In our study, both groups adopted the same hanging system during the treatments, with the shoulder positioned at 90° of anteflexion and relieved of the gravity from both robotic modules and the limb weight. It could be one of the reasons leading to the release of co-contraction patterns, particularly in the proximal arm in the two groups.

There was no adverse event during or after the treatments reported by the trainers and subjects throughout the whole period of this study.

### Limitation

The sample size in this study was small. Despite the relatively small populations recruited, we observed the significant intergroup differences between the two groups by the MAS measurement and EMG parameters. Randomized clinical trials with larger scales (e.g., larger sample sizes and multi-centers) will be conducted to consolidate the rehabilitation effectiveness of the EMG-driven NMES-robot-assisted upper limb training in the future. The design of flexible training frequency, ranging from 3 to 5 sessions/week, was achievable by outpatients with chronic stroke based on our previous experiences [32, 33, 42, 71]. A more constant training frequency for all subjects could be adopted to minimize the possible variation caused by the flexible training frequency in future studies. The co-contraction index between finger flexor and finger extensor did not show significant variations across the twenty training sessions in both groups in this work. It could be related to the evaluation tasks containing the object-hold component by fingers, rather than pure hand open and close motions, which might lead to a high co-contraction between the finger flexor and extensor. The correlation between the co-contraction indexes and FMA scores will be investigated in the future work when the motion tasks for EMG capturing are the same as those in the FMA evaluation.

## Conclusions

In this study, two different supporting schemes for chronic stroke patients were investigated through the UE motor task training assisted by the EMG-driven NMES-robotic systems. According to the results obtained, both schemes supporting either to the distal (i.e. fingers) or to the more proximal (i.e. wrist-elbow) segments could improve the muscle coordination in the entire range of UE motions during daily activities, and the achievements could be maintained for at least three months. The study also indicated that distal support not only led to a similar motor recovery in the proximal UE when compared with direct proximal support but also led to significant better motor recovery in the distal UE than that by proximal support. Furthermore, the distal supporting scheme could effectively release the muscle spasticity in the entire upper limb, especially at the distal UE (i.e. fingers and wrist). The results also suggested that the provision of direct support to the distal joints was more effective than that to the proximal joints in the case of chronic stroke patients.

## Data Availability

The raw data, including the EMG and clinical scores, from individual subjects in the study, cannot be disclosed for public usage. It has been stated in the consent approved by the Human Subjects Ethics Sub-Committee of the Hong Kong Polytechnic University that the results of the experiment may be published, but the individual results should be kept confidentially for each subject.

## Abbreviations

3MFU: 3-month Follow-up
ARAT: Action Research Arm Test
CI: Co-contraction index
EMG: Lectromyography
FMA: Fugl-Meyer Assessment
FMA-SE: Fugl-Meyer Assessment shoulder/elbow sub-scale
FMA-UE: Fugl-Meyer Assessment for upper extremity
FMA-WH: Fugl-Meyer Assessment wrist/hand sub-scale
MAS: Modified Ashworth Scale
MMSE: Mini-Mental State Exam
MVC: Maximum voluntary contraction
NMES: Neuromuscular electrical stimulation
UE: Upper extremity
